# HECTD3 promotes gastric cancer progression by mediating the polyubiquitination of c-MYC

**DOI:** 10.1038/s41420-022-01001-9

**Published:** 2022-04-09

**Authors:** Guanghui Zhang, Qingzong Zhu, Xiaomin Yan, Mingxin Ci, Erhu Zhao, Jianbing Hou, Sicheng Wan, Muhan Lü, Hongjuan Cui

**Affiliations:** 1 State Key Laboratory of Silkworm Genome Biology, Southwest University, 400716 Chongqing, China; 2 Cancer Center, Medical Research Institute, Southwest University, 400716 Chongqing, China; 3 Chongqing iCELL Biotechnology Co Ltd, Chongqing, People’s Republic of China; 4 Department of Gastroenterology, The Affiliated Hospital of Southwest Medical University, 646000 Luzhou, China

**Keywords:** Oncogenes, Cancer therapy

## Abstract

The E3 ubiquitin ligase HECTD3 is homologous with the E6 related protein carboxyl terminus, which plays a vital role in biological modification, including immunoreactivity, drug resistance and apoptosis. Current research indicates that HECTD3 promotes the malignant proliferation of multiple tumors and increases drug tolerance. Our study primarily explored the important function and effects of HECTD3 in gastric cancer. Here, we discovered that HECTD3 is abnormally activated in gastric cancer, and the clinical prognosis database suggested that HECTD3 was strongly expressed in gastric cancer. Depletion of HECTD3 restrained the proliferative and clone abilities of cells and induced the apoptosis of gastric cancer cells. Mechanistically, our findings revealed that interaction between HECTD3 and c-MYC, and that the DOC domain of HECTD3 interacted with the CP and bHLHZ domains of c-MYC. Furthermore, we discovered that HECTD3 mediates K29-linked polyubiquitination of c-MYC. Then, our research indicated that cysteine mutation at amino acid 823 (ubiquitinase active site) of HECTD3 reduces the polyubiquitination of c-MYC. Our experimental results reveal that HECTD3 facilitates the malignant proliferation of gastric cancer by mediating K29 site-linked polyubiquitination of c-MYC. HECTD3 might become a curative marker.

## Introduction

Gastric cancer (GC) is the fifth pernicious tumor worldwide, its incidence and mortality are extraordinarily high [1]. Treatment includes surgery, radiotherapy and chemotherapy; however, the prognosis and overall survival rate of patients remain poor [2]. Therefore, it is particularly urgent to identify therapeutic targets for patients with gastric cancer.

Ubiquitination is a post-translational modification of proteins that is related to many basic biological functions, containing cell cycle, apoptosis, proliferation, signal transduction, cell localization, and the inflammatory response [3, 4]. The E3 ubiquitination ligase HECTD3 is homologous with the carboxyl terminus of E6 related proteins and is classified as the third subfamily of HECT E3s [5]. Over time, the role of HECTD3 in tumors has attracted considerable attention. A recent study indicated that HECTD3 promotes the progression of multiple tumors [6–8]. HECTD3 mediates the non-K48-linked polyubiquitination of STAT3, caspase8, caspase9, MALT1 and TRAF3 [6–9]. However, biological effect of HECTD3 in gastric cancer is unknown. Our results revealed abnormal activation of HECTD3 in gastric cancer cells, and depletion of HECTD3 restrains the proliferative ability of cells and induces apoptosis.

C-MYC was originally authenticated as a cell congenetic retroviruses and an oncogene [10, 11]. Existing studies show that c-MYC is activated in 50% of tumors [12]. Current research indicates that c-MYC acts a significant part in regulating the cell cycle and metabolism, and c-MYC is abnormally activated in a variety of human tumors [13–16]. Consequently, expression of c-MYC is precisely regulated in biological processes. Existing reports indicate that c-MYC contains several domains and that act important roles, including the transaction activation domain (TAD), central portion domain (CP) and basic helix–loop–helix leucine zipper domain (bHLHZ) [17, 18]. Furthermore, C-MYC is a newly discovered ubiquitin target of HECTD3. Our findings discovered that interaction between HECTD3 and c-MYC, and the DOC domain of HECTD3 interacts with the CP and bHLHZ domains of c-MYC. Furthermore, our study determined that HECTD3 mediates K29 site-linked polyubiquitination of c-MYC.

In general, these results revealed that the abnormal activation of HECTD3 in gastric cancer and participates in cell cycle and apoptosis. Mechanistically, our study indicated that interaction between HECTD3 and c-MYC and mediates K29 site-linked polyubiquitination of c-MYC and stabilizes expression of c-MYC. These experimental data reveal that HECTD3 may become a therapeutic marker.

## Results

### Abnormal activation of HECTD3 in gastric cancer leads to poor prognosis for patients

**Fig. 1:**
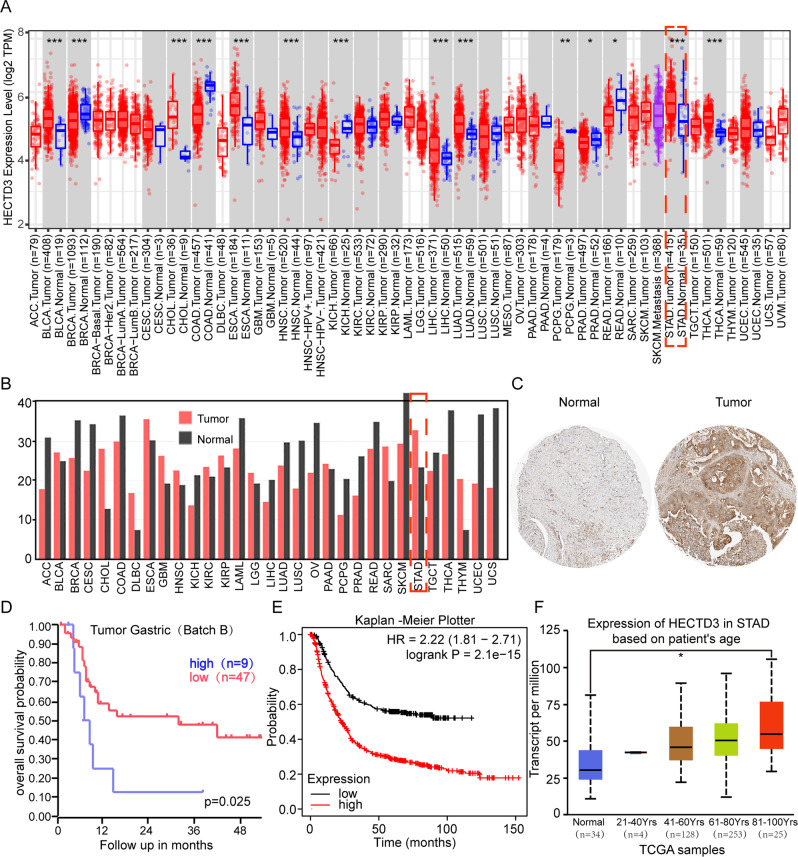
Abnormal activation of HECTD3 in gastric cancer leads to poor prognosis for patients. **A**, **B** Expression of HECTD3 in gastric cancer and many other tumor types was determined using the TIMER2 (http://timer.cistrome.org/) and GEPIA (http://gepia.cancer-pku.cn/index.html) databases. **C** Protein Atlas database (http://www.proteinatlas.org/) was adopted to check expression of HECTD3 in human nontumor gastric mucosa and gastric cancer. **D**, **E** The R2 database and Kaplan–Meier plotter were applied to detect prognostic significance of HECTD3. **F** The expression of HECTD3 was analyzed through the UALCAN database.

To determine the function of HECTD3 in gastric cancer, the expression of HECTD3 was analyzed through TCGA and GEPIA data set. These data set analysis discovered that the overexpression with HECTD3 in gastric cancer and many other tumors (Fig. 1A, B). Subsequently, HECTD3 expression was examined in GES-1 noncancerous gastric mucosa cells and gastric cancer cell lines through western blot. The study indicated that HECTD3 is abnormally activated gastric cancer cells (Supplementary Fig. 1A). Then, the Protein Atlas database [19] was performed to check the positive rate of HECTD3 in human nontumor gastric mucosa and gastric cancer. The database indicated that the expression of HECTD3 significantly increased (Fig. 1C). Next, the prognosis of HECTD3 in gastric cancer was analyzed through the R2 prognosis database and Kaplan–Meier plotter. These experiments indicated that abnormal activation of HECTD3 with poor prognosis in patients (Fig. 1D, E). To further analyze the expression of HECTD3, the age distribution of HECTD3 was assessed in patients with gastric cancer through the UALCAN database. The database analysis indicated that expression of HECTD3 gradually with increasing age in gastric cancer patients (Fig. 1F).

### Depletion of HECTD3 restrains the multiplication of gastric cancer cells

**Fig. 2:**
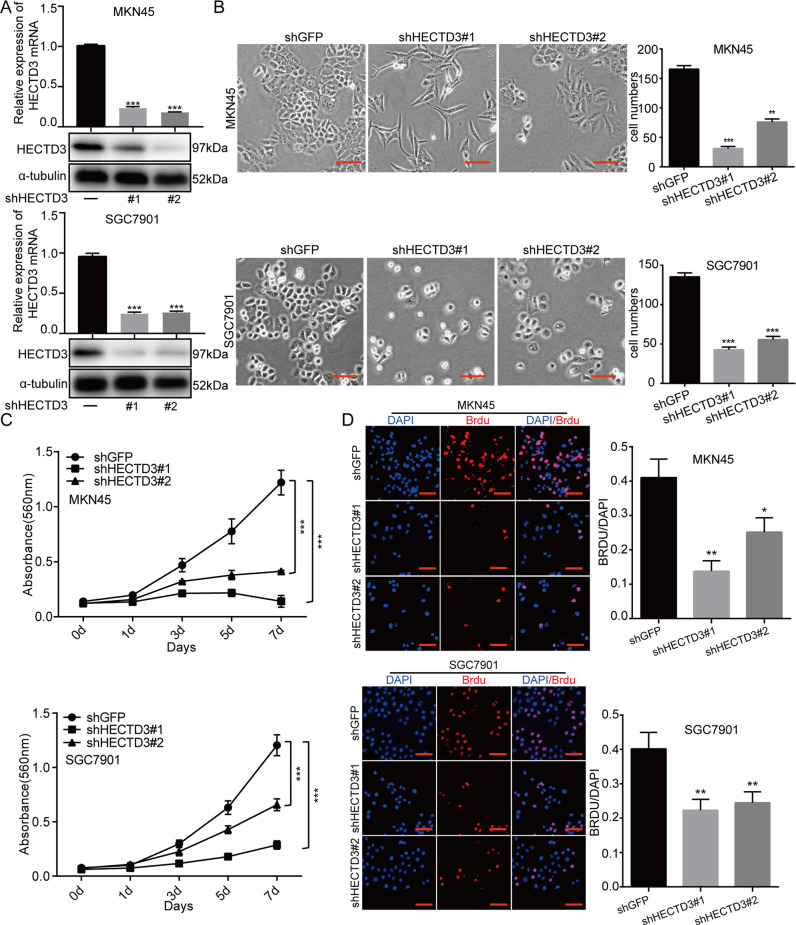
Depletion of HECTD3 restrains the multiplication of gastric cancer cells. **A** Quantitative PCR and western blot were adopted to assess level of HECTD3 with HECTD3-knockdown cells. **B** Morphology and numbers of cells were observed after knocking down HECTD3. **C** MTT curves of HECTD3 knock down gastric cancer cells. **D** Cells positive for BrdU staining are shown after knocking down HECTD3.

To assess influence of HECTD3 on cell multiplication, two small hairpin (sh)RNAs were designed to knock down expression of HECTD3 (Supplementary table). Quantitative PCR and western blot assays were employed to disclose level of HECTD3 in MKN45 and SGC7901 cells (Supplementary table). Experimental data demonstrated that expression of HECTD3 was successfully knocked down (Fig. 2A). Then, the cells with knock down of HECTD3 exhibited marked morphological differences and the number of cells was significantly decreased (Fig. 2B). Furthermore, an MTT experiment was applied to determine the proliferation abilities of cells. The results indicated that depletion of HECTD3 clearly restrained the multiplication ability of gastric cancer cells (Fig. 2C). Next, BrdU incorporation experiment was used to assess DNA synthesis, and the results indicated that the DNA synthesis was significantly reduced in HECTD3 knock down cells (Fig. 2D). Our experimental data suggested that HECTD3 is essential for gastric cancer cell multiplication.

### Depletion of HECTD3 blocks the cell cycle and induces cell apoptosis

**Fig. 3:**
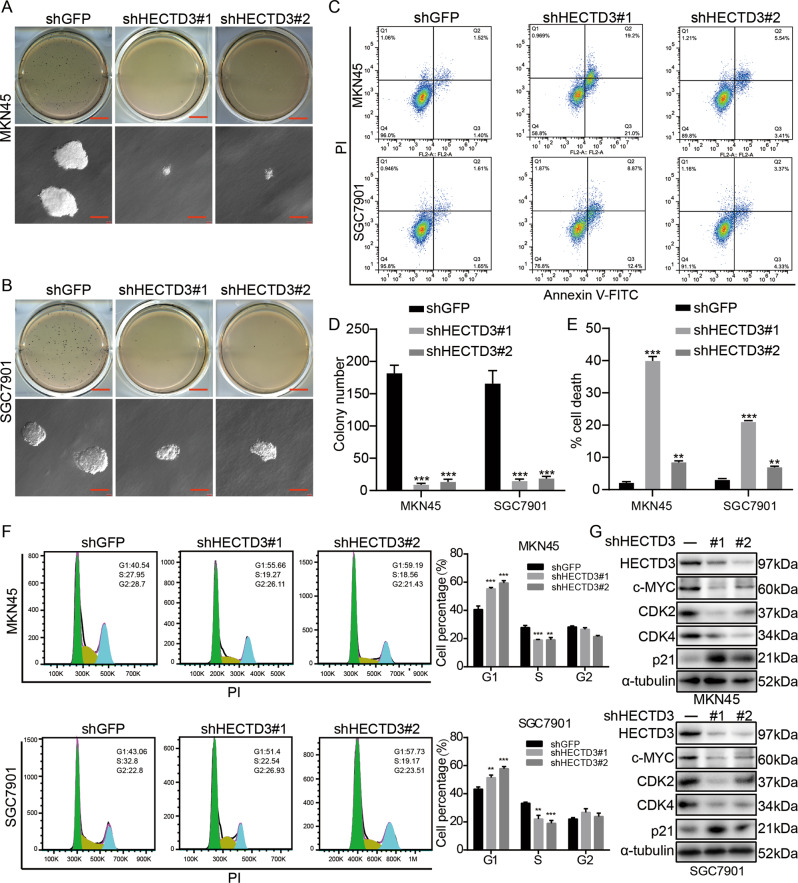
Depletion of HECTD3 blocks the cell cycle and induces cell apoptosis. **A**, **B**, **D** Clone-forming ability was observed in knocking down HECTD3 cells. **C**, **E** Apoptosis of gastric cancer cells with HECTD3-knockdown was analyzed using flow cytometry. **F** The cell cycle was analyzed in HECTD3-knock down gastric cancer cells through flow cytometry. **G** Western blot was applied to check the protein level of HECTD3, c-MYC, CDK2, CDK4, p21, and α-Tubulin in HECTD3 knock down cells.

To evaluate effect of HECTD3 on clone-forming capability, Soft-agar experiment was used to analyze the amount and dimensions of clones in HECTD3-knock down cells. These experiments discovered that the amount and dimensions of clones in HECTD3-knock down cells were obviously decreased (Fig. 3A, B, D). Then, flow cytometry experiment was adopted to check the function of HECTD3 on cell cycle and apoptosis. The experimental data discovered that cell cycle was obviously arrested in G1 period and induced apoptosis of cells in response to knock down of HECTD3 (Fig. 3C, E, F). Furthermore, western blot experiments were used to assess cycle-related proteins. Our experimental data exhibited that the level of c-MYC, CDK2 and CDK4 were markedly reduced while protein expression of p21 was significantly increased in HECTD3 knock down cell lines (Fig. 3G). These assay data showed that HECTD3 played a crucial part in cell cycle and apoptosis.

### HECTD3 is essential for gastric cancer cell-proliferative ability

**Fig. 4:**
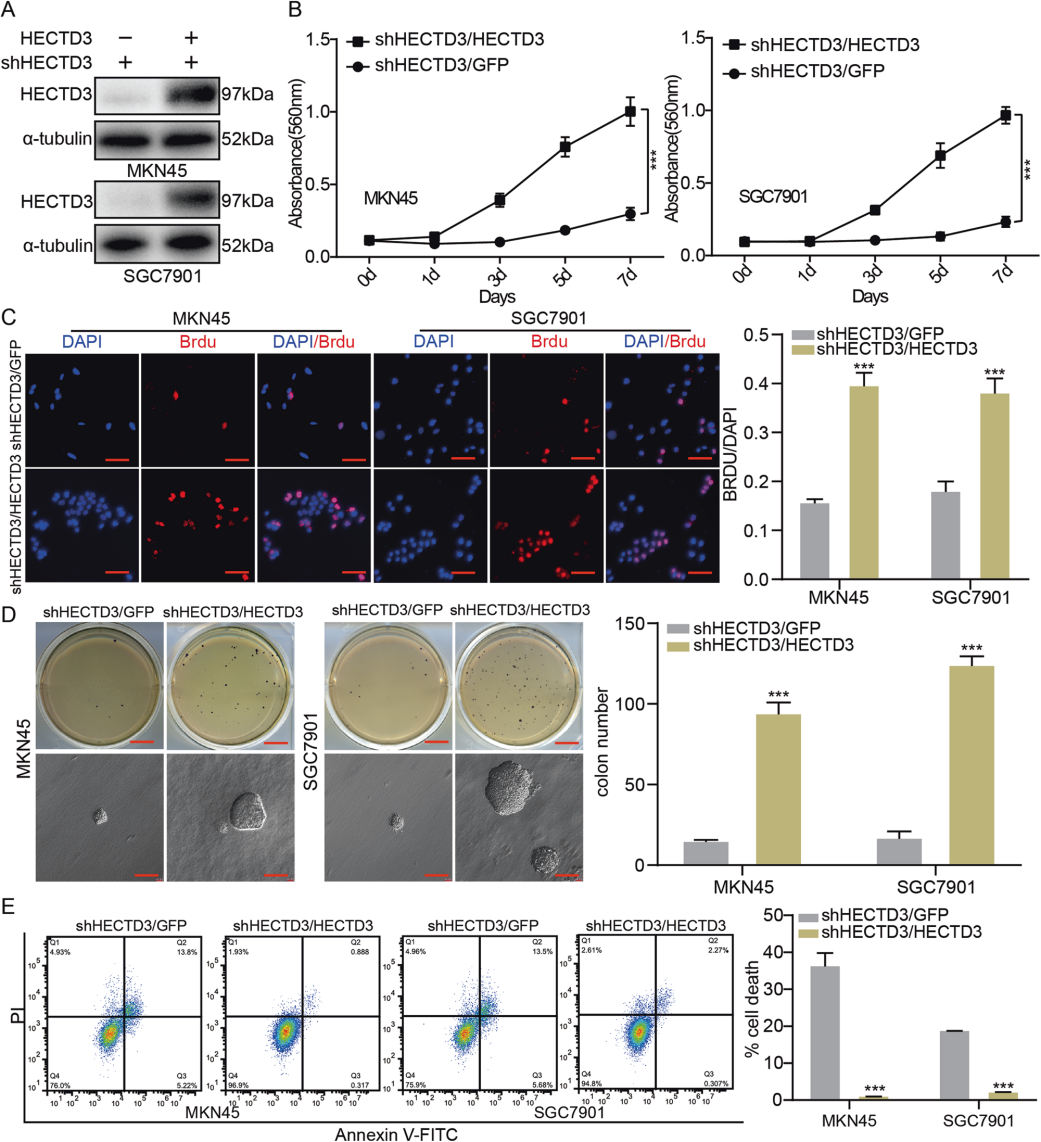
HECTD3 is essential for gastric cancer cell-proliferative ability. **A**, **B** Western blot and MTT was used to examine expression of HECTD3 and proliferation of cells in response to overexpression of HECTD3 in HECTD3-knockdown gastric cancer cell. **C** Cells positive for BrdU staining after overexpression of HECTD3 in HECTD3-knockdown gastric cancer cells are shown. **D**, **E** Colony-formation ability and apoptosis of HECTD3-overexpressing HECTD3 cells in HECTD3-knockdown gastric cancer cells.

To verify that the restrain of cell multiplication caused through HECTD3 knock down was not caused by off-target effects, expression of HECTD3 was rescued in HECTD3 knockdown cells. Western blot and MTT experiments were used to determine proliferation of HECTD3-overexpressing HECTD3-knockdown gastric cancer cells. These assay data discovered that proliferative abilities of cells were obviously rescued (Fig. 4A, B). Then, BrdU experiments were adopted to determine the levels of DNA synthesis in response to overexpression of HECTD3 in HECTD3 knock down gastric cancer cells, and the experiments revealed that levels of DNA synthesis were significantly rescued compared to that in the control groups (Fig. 4C). Furthermore, soft-agar experiment was applied to test the number and size of clones after overexpression of HECTD3 in knocking down HECTD3 cells and these experimental data suggested that the amount and dimension of clones were partially rescued compared with those of the controls (Fig. 4D). Next, flow cytometry assay was applied to analyze the influence of HECTD3 on apoptosis after in response to overexpression of HECTD3 in HECTD3-knockdown cells. These research results discovered that the apoptosis of HECTD3-knock down cells was rescued after overexpression of HECTD3 (Fig. 4E). Our study indicated that HECTD3 is essential for gastric cancer cell multiplication.

### HECTD3 interacted with c-MYC and mediates K29 site-linked polyubiquitination of c-MYC

**Fig. 5:**
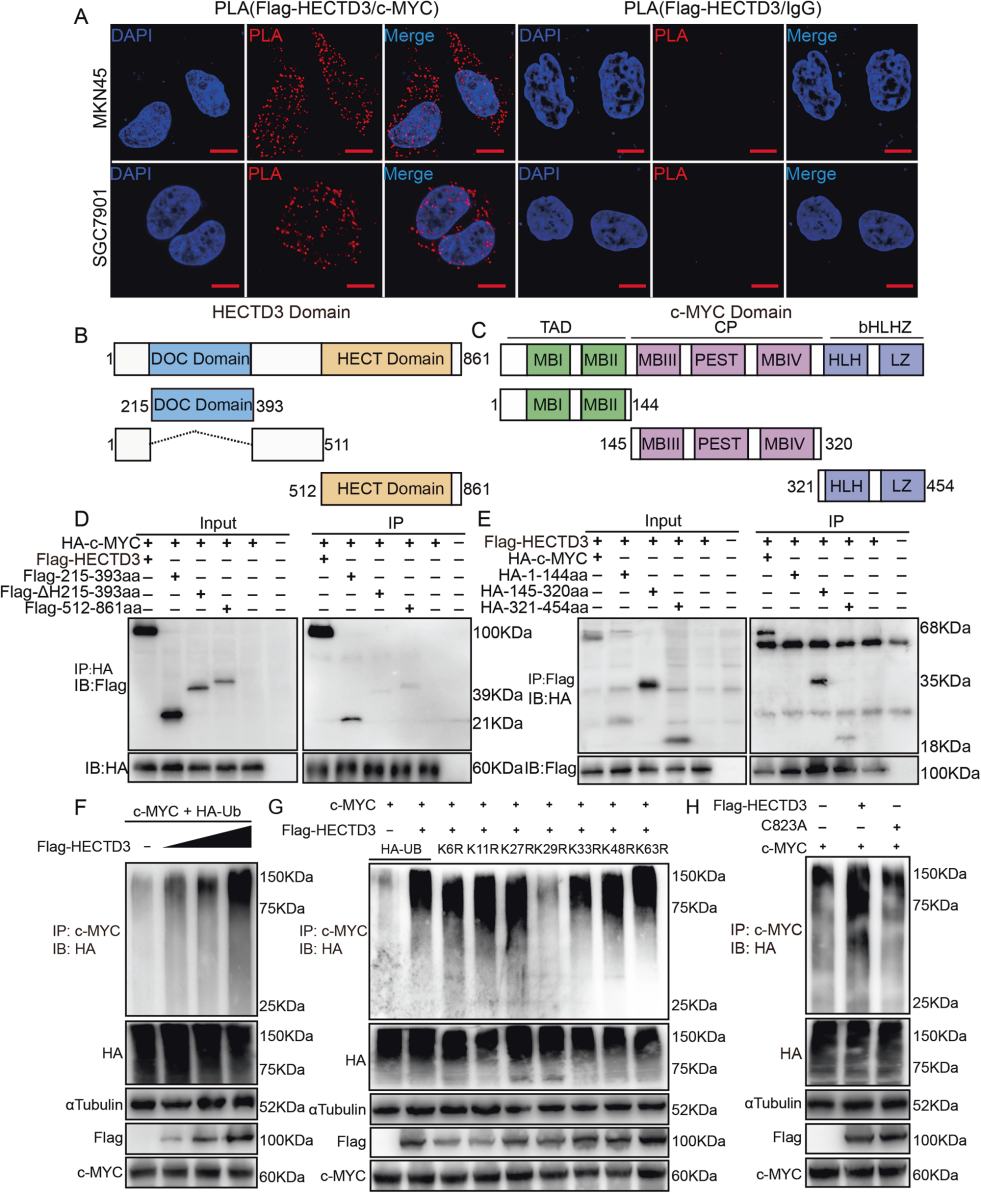
HECTD3 interacts with c-MYC and mediates K29 site-linked polyubiquitination of c-MYC. **A** The proximity ligation (PLA) experiments were applied to assess the interaction of HECTD3 and c-MYC. **B** DOC and HECT domains diagram of HECTD3. **C** TAD, CP, and bHLHZ domains diagram of c-MYC. **D**, **E** Interaction between c-MYC and HECTD3 truncated domains in 293FT cells. **F** 293FT cells were cotransfected with c-MYC, HA-Ub, and increasing amounts of HECTD3 plasmids and treated with MG132 were immunoprecipitated using PARP1 beads and immunoblotted using an anti-HA antibody. **G** The Flag-HECTD3, c-MYC, HA-Ub, and ubiquitin mutant plasmid were cotransfected and treated with MG132 for ubiquitination assays in 293FT cells. **H** The Flag-HECTD3, c-MYC, HA-Ub, and C823A were cotransferred and treated with MG132 for ubiquitination assay in 293FT cells.

To make efforts investigate mechanism of HECTD3 in gastric cancer. Then, we detected the proteins interacting with HECTD3 using the database, and the database results indicated that interaction between HECTD3 and c-MYC. Next, a proximity ligation (PLA) experiment was employed to observe the interaction of HECTD3 and c-MYC in gastric cancer cells. The assay data revealed that HECTD3 interacts with c-MYC (Fig. 5A and Supplementary Fig. 2A). Previous studies have shown that HECTD3 contains DOC domain and HECT domain, while c-MYC contains TAD, CP and bHLHZ domains. To further verify the interaction between HECTD3 and c-MYC. Flag-tagged DOC domain (215–393aa), ΔH (215–393aa) region, HECT domain (512–861aa) and full-length Flag-tagged HECTD3 plasmids were constructed for transfection experiments. Then, HA-tagged TAD domain (1–144aa), CP domain (145–320aa) and bHLHZ domain (321–454aa) plasmids were constructed for transfection experiments (Fig. 5B, C). Immunoprecipitation (IP) assays were performed to use lysates prepared from HEK293 transiently transfected with Flag-tagged DOC domain (215–393aa), ΔH (215–393aa) region, HECT domain (512–861aa), full-length Flag-tagged HECTD3, HA-tagged TAD domain (1–144aa), CP domain (145–320aa), bHLHZ domain (321–454aa) plasmids and full-length HA-tagged C-MYC plasmids. The results indicated that the DOC domain of HECTD3 interacts with the CP and bHLHZ domains of c-MYC (Fig. 5D, E). Recent studies reported that HECTD3 mediates the non-K48-linked polyubiquitination of STAT3, caspase8, caspase9, MALT1 and TRAF3. We speculated that HECTD3 regulates the polyubiquitination of c-MYC. Then, the mRNA of c-MYC was detected through fluorescence quantitative PCR. Our assay results discovered that the mRNA levels of c-MYC was no markedly change in knockdown HECTD3 gastric cancer cells (Supplementary Fig. 3C). Furthermore, proteasome inhibitor MG132 was added to gastric cancer cells in knockdown HECTD3 cells, and the experimental data displayed that the expression of c-MYC was partially restored (Supplementary Fig. 3D). Then, ubiquitination experiment was applied in vitro and the experimental data discovered that HECTD3 enhanced the ubiquitination of c-MYC (Fig. 5F). The constructed ubiquitination plasmids, Flag-HECTD3 and c-MYC were cotransfected into 293FT cells for ubiquitination assays and the results showed that HECTD3 mediates K29-linked polyubiquitination of c-MYC (Fig. 5G). It has been reported that cysteine at 823 of HECTD3 plays an important role in polyubiquitination. Then, Flag-tagged HECTD3 mutant plasmid (C823A), HECTD3, c-MYC and HA-Ub were cotransfected into 293FT cells for ubiquitination assay. The assay results suggested that polyubiquitination of c-MYC was markedly decreased after cysteine mutation at 823 (ubiquitinase active site) of HECTD3, compared to the wild type (Fig. 5H). These data revealed that HECTD3 mediated K29-linked polyubiquitination of c-MYC. Existing studies have shown that the reduction of k29-linked polyubiquitination leads to cell cycle arrest [20]. Our data discovered that HECTD3 mediated K29-linked polyubiquitination of c-MYC and regulated the cell cycle and apoptosis. Furthermore, Cycloheximide (CHX) was added to overexpressed HECTD3 cells. The results suggested that the degradation rate of c-MYC slowed down (Supplementary Fig. 3A, B). These data suggested that HECTD3 stabilized expression of c-MYC through mediating K29-linked polyubiquitination of c-MYC.

### HECTD3 is required for tumorigenesis of gastric cancer cells

**Fig. 6:**
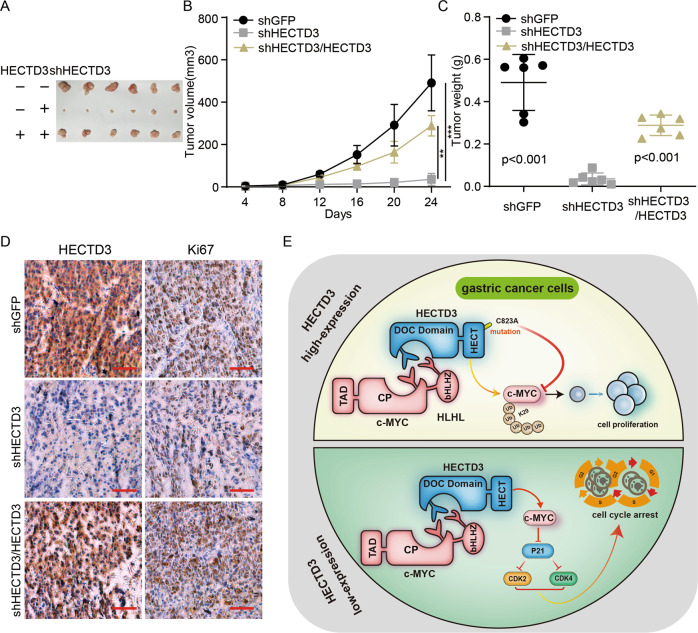
HECTD3 is required for tumorigenesis of gastric cancer cells. **A**–**C** The size, growth curve, and weight of tumor were measured in knocking down HECTD3 and HECTD3-rescued knockdown HECTD3 gastric cancer cells. **D** Expressions of HECTD3 and Ki67 was detected by immunohistochemistry in response to overexpression of HECTD3 in HECTD3-knockdown tumor tissues. **E** The proposed model and mechanistic diagram through which HECTD3 regulates c-MYC.

To assess influence of HECTD3 on the oncogenesis of cells in vivo, subcutaneous tumor formation experiments were performed in mice and the assay results manifested that the growth curve, volume and weight of subcutaneous tumor in knocking down HECTD3 gastric cancer cells were clearly reduced (Fig. 6A, B, C). Immunohistochemical staining assays indicated that the positive rates of HECTD3 and Ki67 were decreased in HECTD3-knock down tumors. Then, in response to overexpression of HECTD3 in HECTD3 knock down cells, oncogenesis was partially rescued, and the positive rate of HECTD3 and Ki67 was also restored (Fig. 6D).

## Discussion

Gastric cancer (GC) is the fifth pernicious tumor worldwide, and its incidence and mortality are extraordinarily high [21, 22]. Studies have shown that Helicobacter pylori are the primary risk factor for gastric cancer, and almost all cases of gastric cancer are related to Helicobacter pylori [23]. In addition, there are other factors, such as gender, age, diet, ethnic background and that play a vital role in occurrence of GC [24]. Treatment of patients with gastric cancer includes surgery, radiotherapy and chemotherapy, however, the prognosis and 5-year overall survival of patients remain poor [2]. Although gastric cancer has been widely studied, its potential molecular mechanism is still elusive, so it is more urgent to identify biomarkers of gastric cancer. Current research indicates that HECTD3 is abnormally activated in many types of tumors and functions an oncogene. In our assay, we explored the function and role of HECTD3 in gastric cancer. Depletion of HECTD3 restrained the proliferative ability of cells and blocked the cell cycle at G1 phase. These data indicated that HECTD3 may represent a therapeutic target in patients with gastric cancer.

C-MYC is act an oncogene and that also serves as a transcription factor [25]. Existing research reveals that c-MYC plays crucial part in cell proliferation, apoptosis, angiogenesis and metabolism. Thence, the c-MYC is rigorously regulated in biological processes [26–28]. To date, a variety of ubiquitination ligases and deubiquitination enzymes have been found to participate in the ubiquitination of c-MYC, and then regulate the activity of c-MYC [15]. HECTD3 mediates the non-K48-linked polyubiquitination of STAT3, caspase8, caspase9, MALT1 and TRAF3 [6–9]. Our study indicated that HECTD3 interacts with c-MYC, and the DOC domain of HECTD3 interacted with the CP and bHLHZ domains of c-MYC. Our results identify c-MYC as a newly discovered HECTD3-binding protein. Our data revealed that HECTD3 mediates K29-linked polyubiquitination of c-MYC. Here, we determined that HECTD3 mediated polyubiquitination of K29 on c-MYC offset the degradation of c-MYC by other ubiquitinases. These results need to be further explored. These assay data suggested that the study of HECTD3 in gastric cancer warrants further attention.

In this study, our results showed that interaction between HECTD3 and c-MYC, and the DOC domain of HECTD3 combines with the CP and bHLHZ domains of c-MYC. Next, we discovered that HECTD3 mediates K29 site-linked polyubiquitination of c-MYC. Furthermore, our study indicated that cysteine mutation at 823 (ubiquitinase active site) of HECTD3 reduces the polyubiquitination of c-MYC. Therefore, we speculated that targeting drugs targeting cysteine at the 823 site of HECTD3 may become a therapeutic target spot. In general, based on the abnormal expression of HECTD3 in a variety of tumors, HECTD3 may represent a powerful target for a variety of cancer treatments and bringing much needed relief to human tumor patients.

## Materials and methods

### Cell culture

Cell lines (SGC7901, HGC27, MKN45, and MGC803) and gastric mucosa cell lines GES-1 were bought from American Type Culture Collection. 293FT cell lines were bought from Invitrogen^TM^. All cells were fostered by the method described earlier [29].

### Reagents and antibodies

MG132 and Liposome 2000 were obtained from Sigma (Shanghai, China). Immunohistochemical kits were obtained from ZSGB-Bio (Beijing, China). Antibodies against HECTD3 (11487-1-AP), CDK2 (10122-1-AP), CDK4 (11026-1-AP), Tubulin (11224-1-AP), HA-tag (51064-2-AP), p21 (10355-1-AP) and C-MYC (10828-1-AP) were bought through Proteintech. HECTD3 antibody (bs-15448R) was obtained from Bo Aosen Biotechnology Co., Ltd (Beijing, China) for immunohistochemistry (IHC). Antibodies Flag and Ki67 antibodies were obtained through Sigma-Aldrich (Shanghai, China).

### Plasmids, transfection, and infection experiments

HECTD3 small hairpin shRNAs (shHECTD3#1, shHECTD3#2) were cloned into pLKO.1 lentiviral vectors. The recombinant plasmids containing human Flag-HECTD3, Flag-HECTD3 (C823A), Flag-HECTD3-1-511 (amino acids ΔH215–393), HA-c-MYC (1–454), and c-MYC (1–454) were acquired from Youbao Company (Changsha, China). Flag-HECTD3 (amino acids 512–861), Flag-HECTD3 (amino acids 215–393), HA-c-MYC (amino acids 1–144), HA-c-MYC (amino acids 145–320), and HA-c-MYC (amino acids 321–454) were structured into the pCDH-CMV-MCS-EF1-GFP-Puro. Ubiquitination plasmids that contained HA-Ub (K6R, K11R, K27R, K29R, K33R, K48R, and K63R) and Ub-WT were cloned into the pCDH vector and pRK5-HA, which were obtained from Youbao Corporation (Changsha, China). Finally, the plasmids were transferred into cells for follow-up experiments.

### Immunohistochemistry staining

For immunohistochemistry staining analysis, according to previous experimental methods [29], HECTD3 and Ki67 antibodies were diluted in phosphate-buffered saline, and incubated on tumor tissue sections at 4 °C. Finally, immunohistochemical secondary antibody was added for incubation, followed by DAB staining, hematoxylin re-staining and imaging.

### Fluorescence quantitative PCR

TRIzol reagent was applied to extract cellular RNA, and then reverse transcribed into cDNA for subsequent experiments.

### Cell-viability assay

The MTT experiment was used to determine cell proliferation ability as previously assay [29].

### BrdU staining

According to previous experimental methods [29], Cells plated 24-well plates were fixed in paraformaldehyde, BrdU primary antibody was incubated (Abcam, Cambridge, MA, USA) with cells overnight. Finally, nuclei were stained by DAPI, and followed by imaging to select the field of vision.

### Flow cytometry

Cells were gathered and stained through cell cycle and apoptosis kits. Finally, cells were analyzed through flow cytometry.

### Western blot assay

Cells lysis buffer was used to extract proteins from cells. Then, proteins were separated through polyacrylamide gel electrophoresis. Next, antibody was sequentially incubated with membranes, and finally analyzed using a WB detection system.

### Soft-agar assay

Agarose medium (0.6%) was put into the lower layer of the 6-well plates, and then 0.3% agarose medium containing 800 cells was put into the on top. Next, it was placed in the incubator for about 20 days. Finally, the treatment was microscopically observed.

### IP assay

According to previous experimental methods [30]. IP lysate was used to lyse the cells and the primary antibody and protein A/g magnetic beads were selected for incubation, beads were washed, and finally the cells were denatured in a 100 °C water bath for subsequent western blot experiments.

### Ubiquitination assay

Flag-HECTD3, Flag-HECTD3 (C823A), C-MYC, and ubiquitination plasmids were transfected into 293FT cells through Liposome 2000. Two days later, the proteasome inhibitor MG132 was put into the cells for 7 h. Finally, the cells were gathered for subsequent IP and WB experiments.

### Animal assay

The mice experiment was agreed through the animal ethics committee of Southwest University and followed the animal health guidelines. Female nude mice were obtained from Huafukang Biotechnology Co., Ltd. Then, the nude mice were stochastically distributed into three groups (6/group). Mice were anesthetized using an isoflurane nasal anesthesia system to reduce the pain caused by subcutaneous injection. Cells (MKN45) (1 × 10^6^ cells) stably transfected with shGFP, shHECTD3 and shHECTD3/HECTD3 were squirted into the subcutaneous of nude mice. The mice were observed every four days after subcutaneous injection. Before collecting the tumor, the mice were anesthetized using an isoflurane nasal anesthesia system. Finally mice were euthanized through cervical dislocation and mice corpses were collected and stored at –20 °C and then sent to laibit Biotechnology Co., Ltd. (Chongqing, China) for incineration. Then, tumor volumes were calculated through *V* = (length × width^2^)/2. Finally, immunohistochemical experiment was performed.

### Patient database analysis

All data sets were obtained from GEPIA (http://gepia.cancer-pku.cn/index.html) databases, TIMER2 (http://timer.cistrome.org/), R2 databases (https://hgserver1.amc.nl/cgi-bin/r2/main.cgi), gastric cancer (Kaplan–Meier Plotter) databases and Protein Atlas database (http://www.proteinatlas.org/).

### Statistical analysis

All samples are based on a two tailed Student’s *t*-test. *P* < 0.05 was considered statistically significant.

## Supplementary information

Supplementary Figure 1

Supplementary Figure 2

Supplementary Figure 3

Supplementary Figure notes

Supplementary Table

cddiscovery-author-contribution-form

aj-checklist

Raw data-BrdU and White light diagram

Raw data-cell cycle and apoptosis

Raw data-PLA assay

Raw data-Western blot

Raw IHC and Xenograft experiment

## Data Availability

All data and material in the study are available when requested.
