# Supplementary figures and images for: HECTD3 promotes gastric cancer progression by mediating the polyubiquitination of c-MYC
Source: Cell Death Discov. 2022 Apr 9;8:185. doi: 10.1038/s41420-022-01001-9 (PMC8994759; doi:10.1038/s41420-022-01001-9)

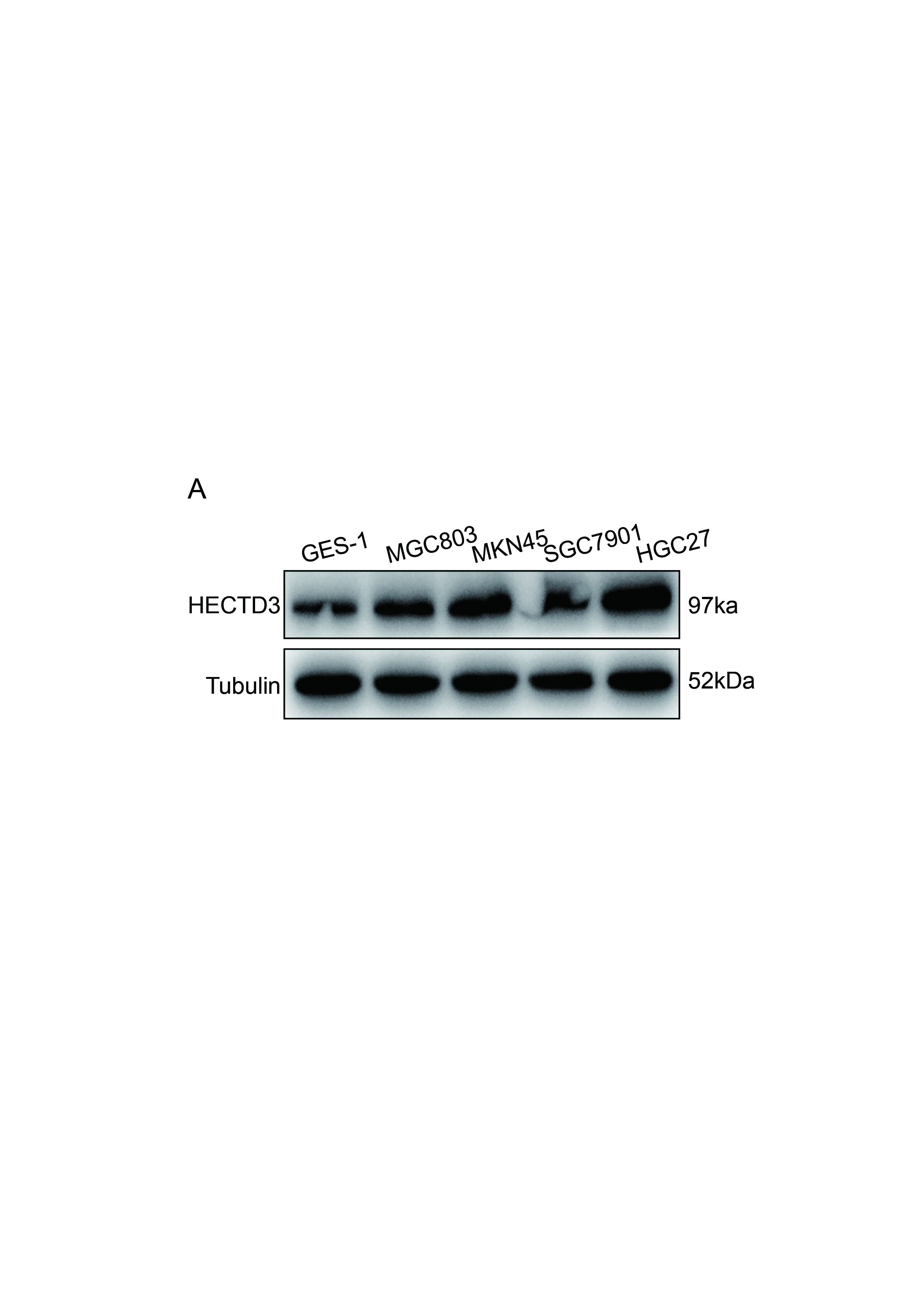

Supplement: Supplementary file 1 — Supplementary Figure 1 [file 41420_2022_1001_MOESM1_ESM.tif]

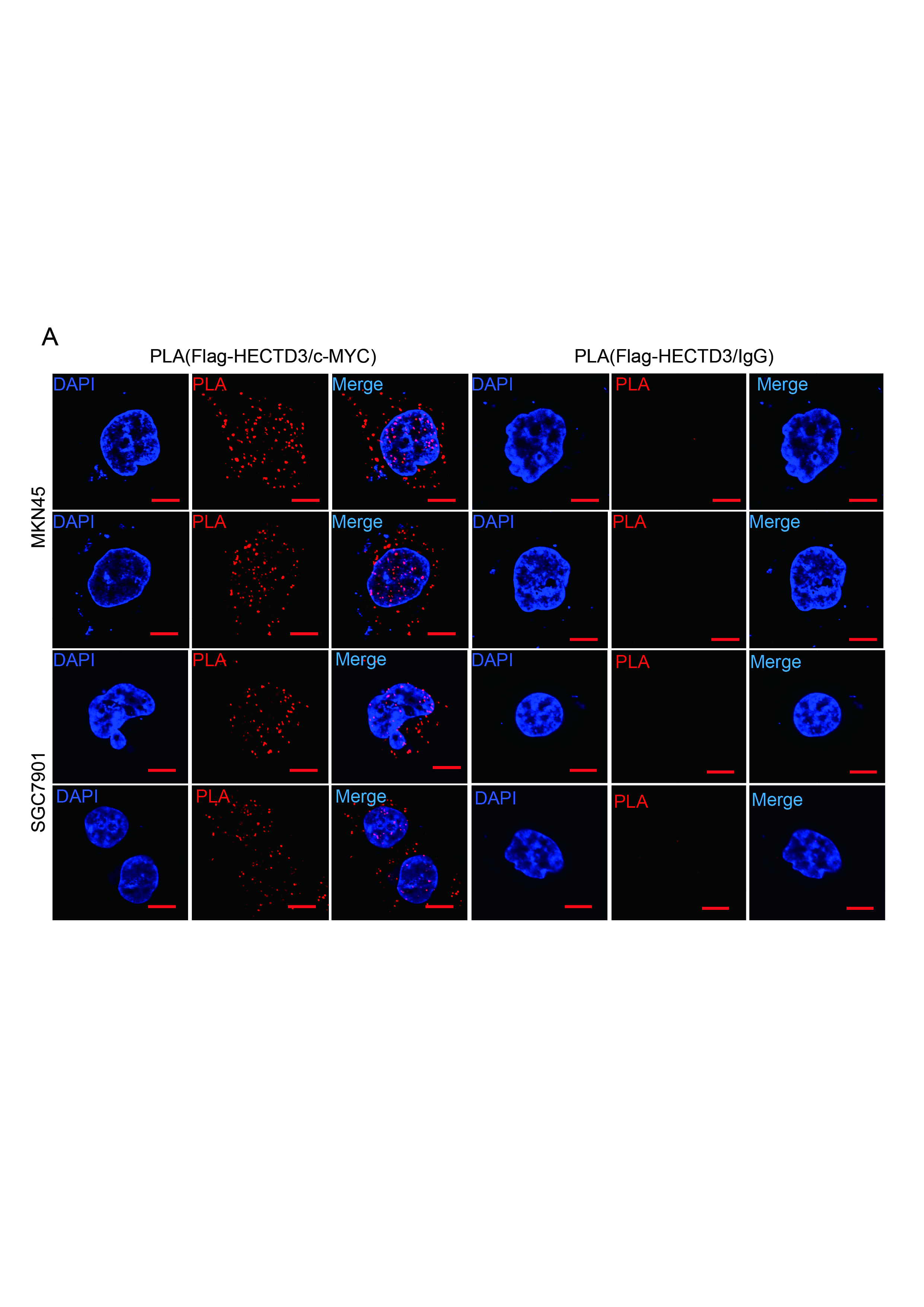

Supplement: Supplementary file 2 — Supplementary Figure 2 [file 41420_2022_1001_MOESM2_ESM.tif]

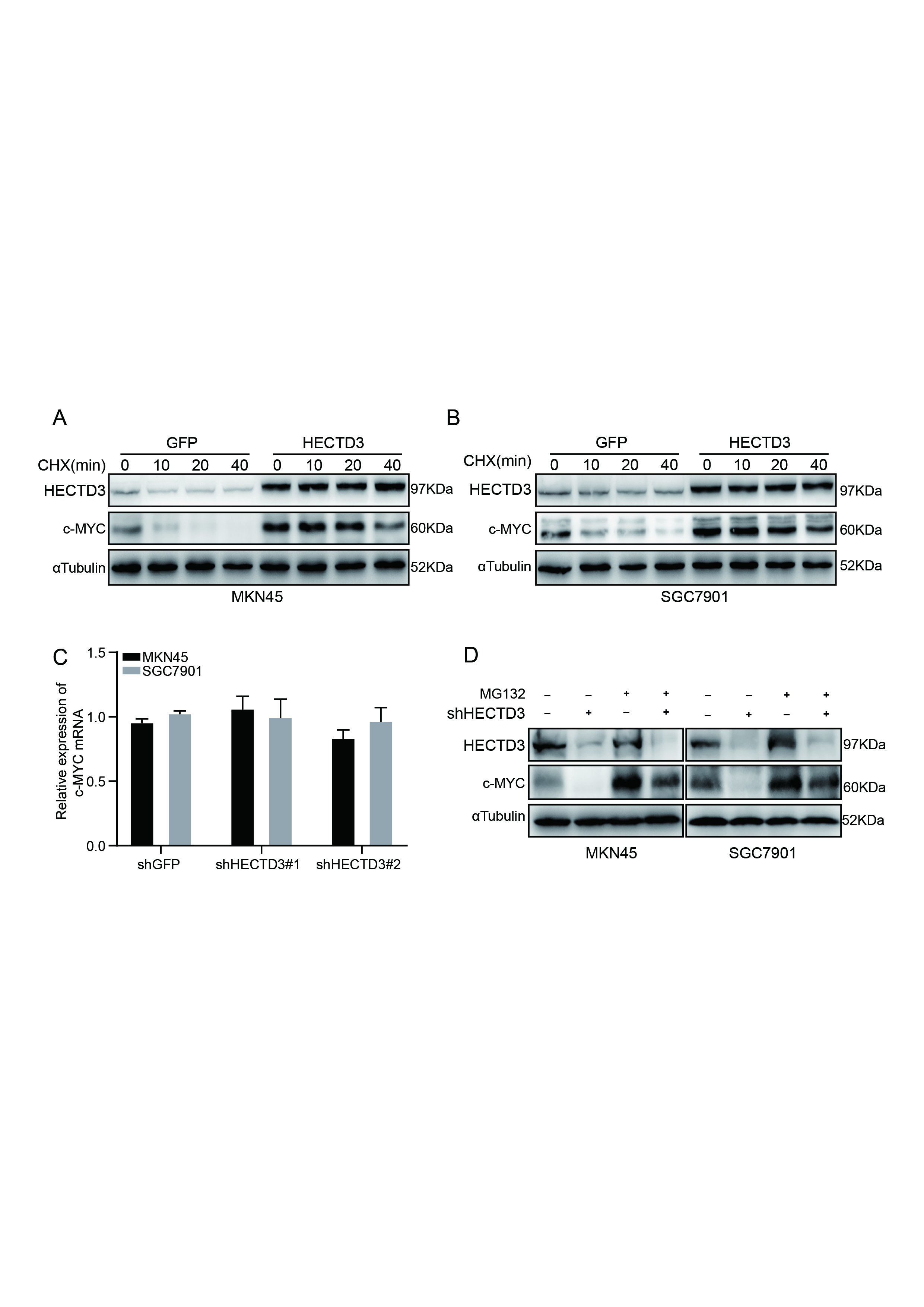

Supplement: Supplementary file 3 — Supplementary Figure 3 [file 41420_2022_1001_MOESM3_ESM.tif]

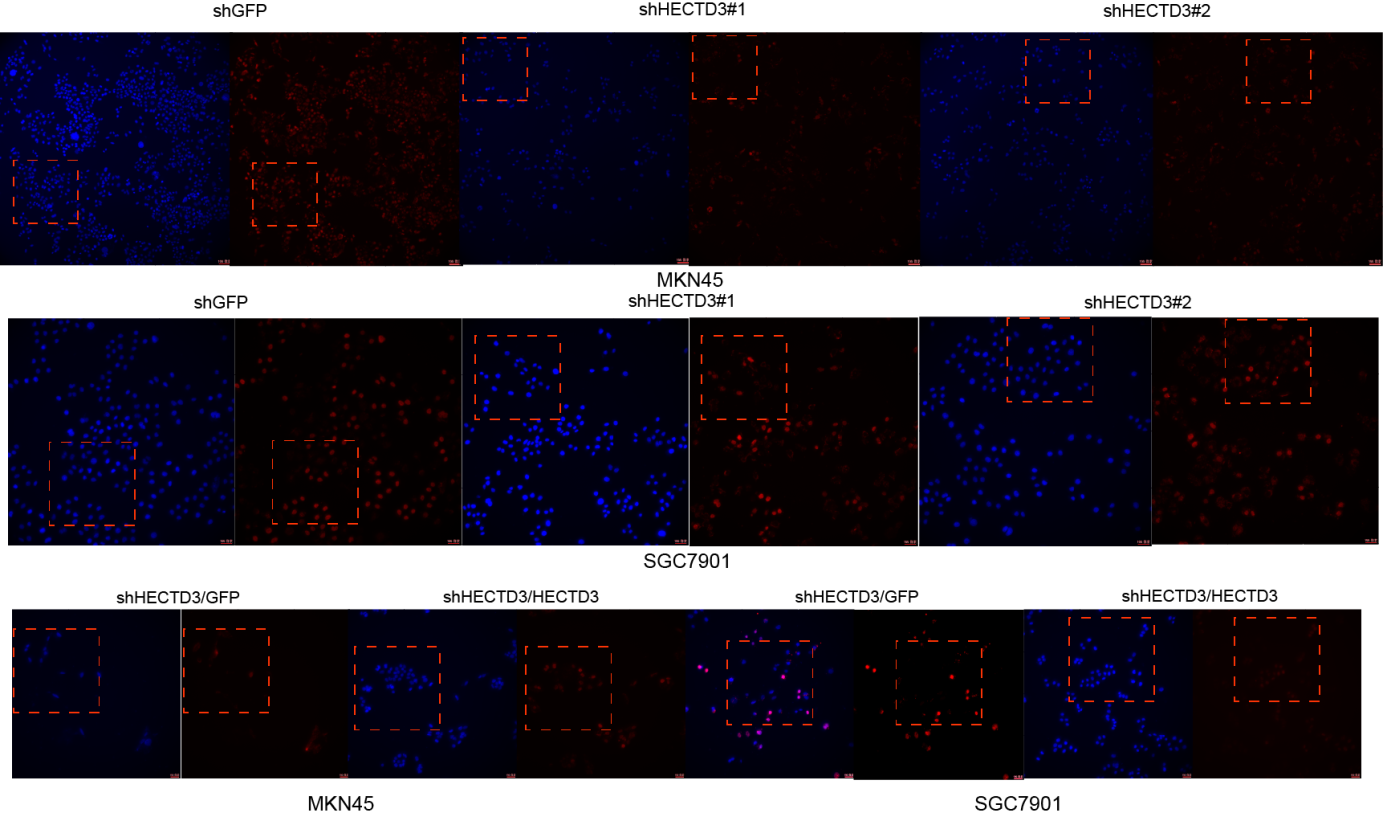

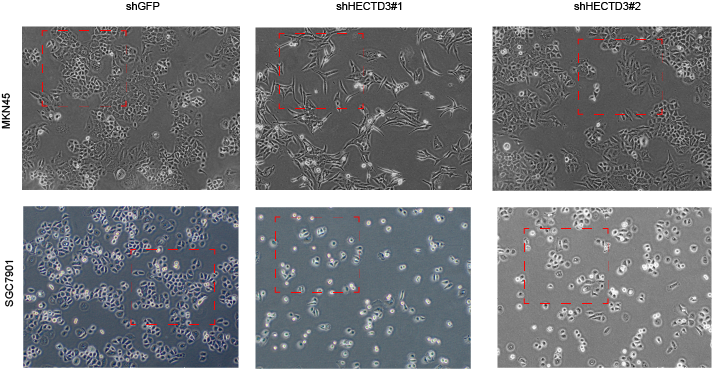

Supplement: Supplementary file 8 — Raw data-BrdU and White light diagram [file 41420_2022_1001_MOESM8_ESM.docx]

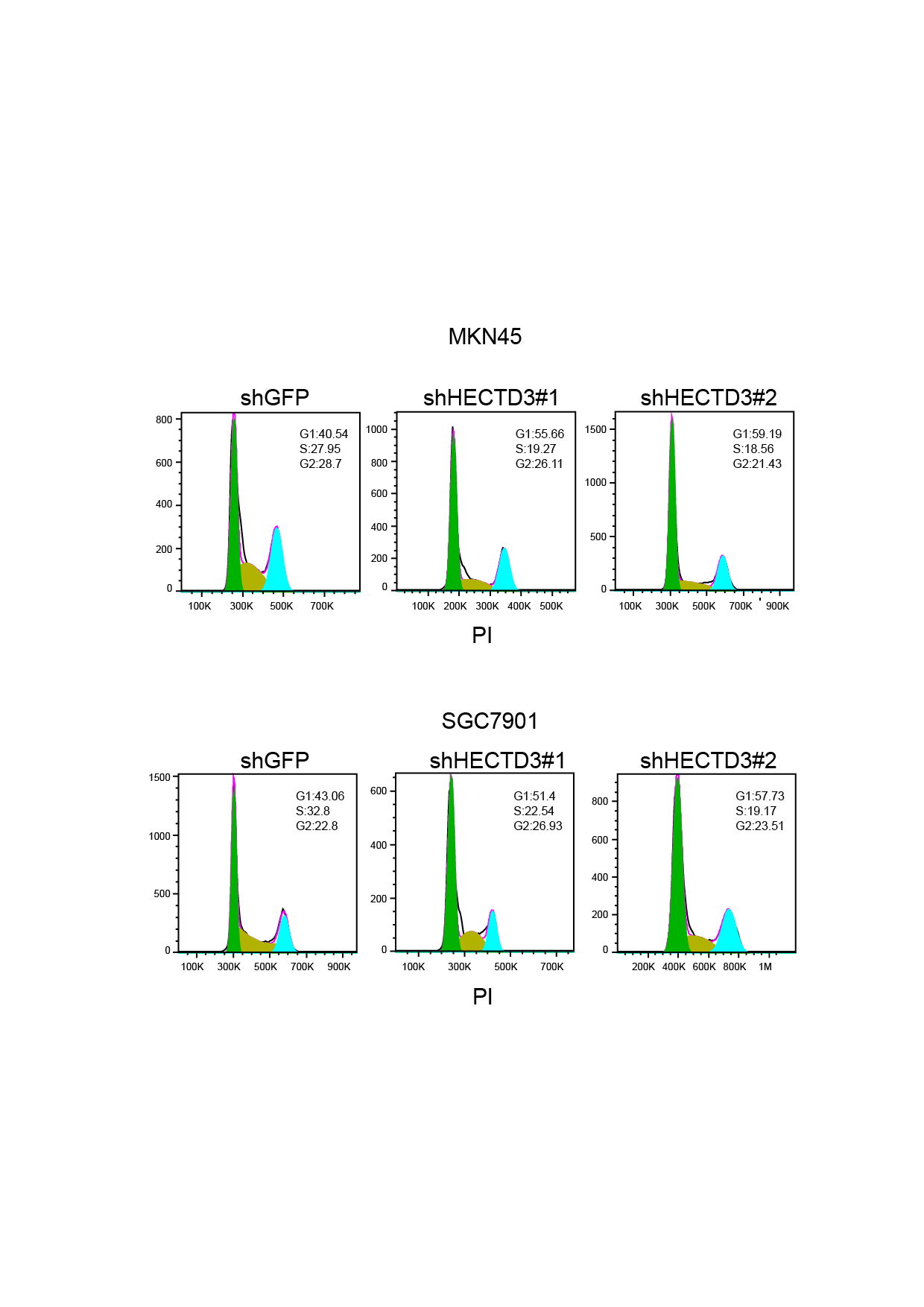


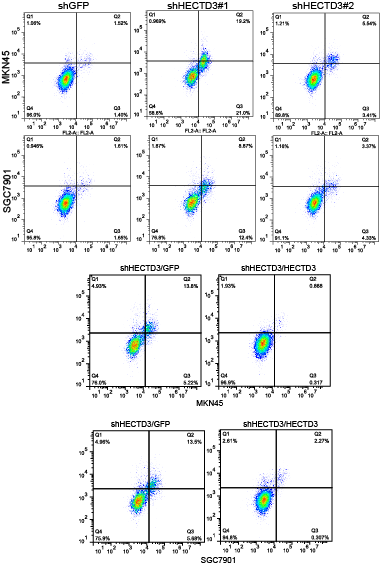

Supplement: Supplementary file 9 — Raw data-cell cycle and apoptosis [file 41420_2022_1001_MOESM9_ESM.docx]

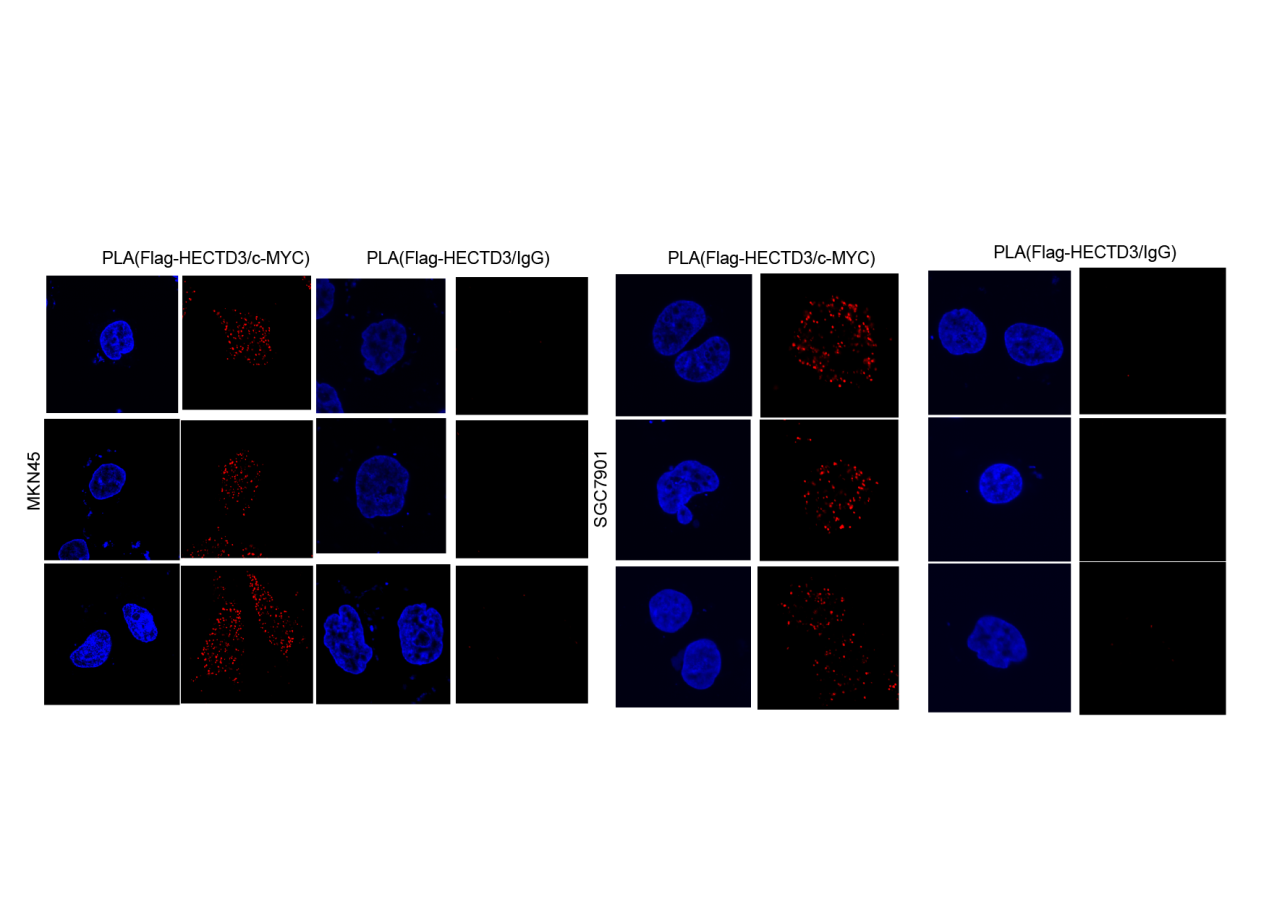

Supplement: Supplementary file 10 — Raw data-PLA assay [file 41420_2022_1001_MOESM10_ESM.docx]

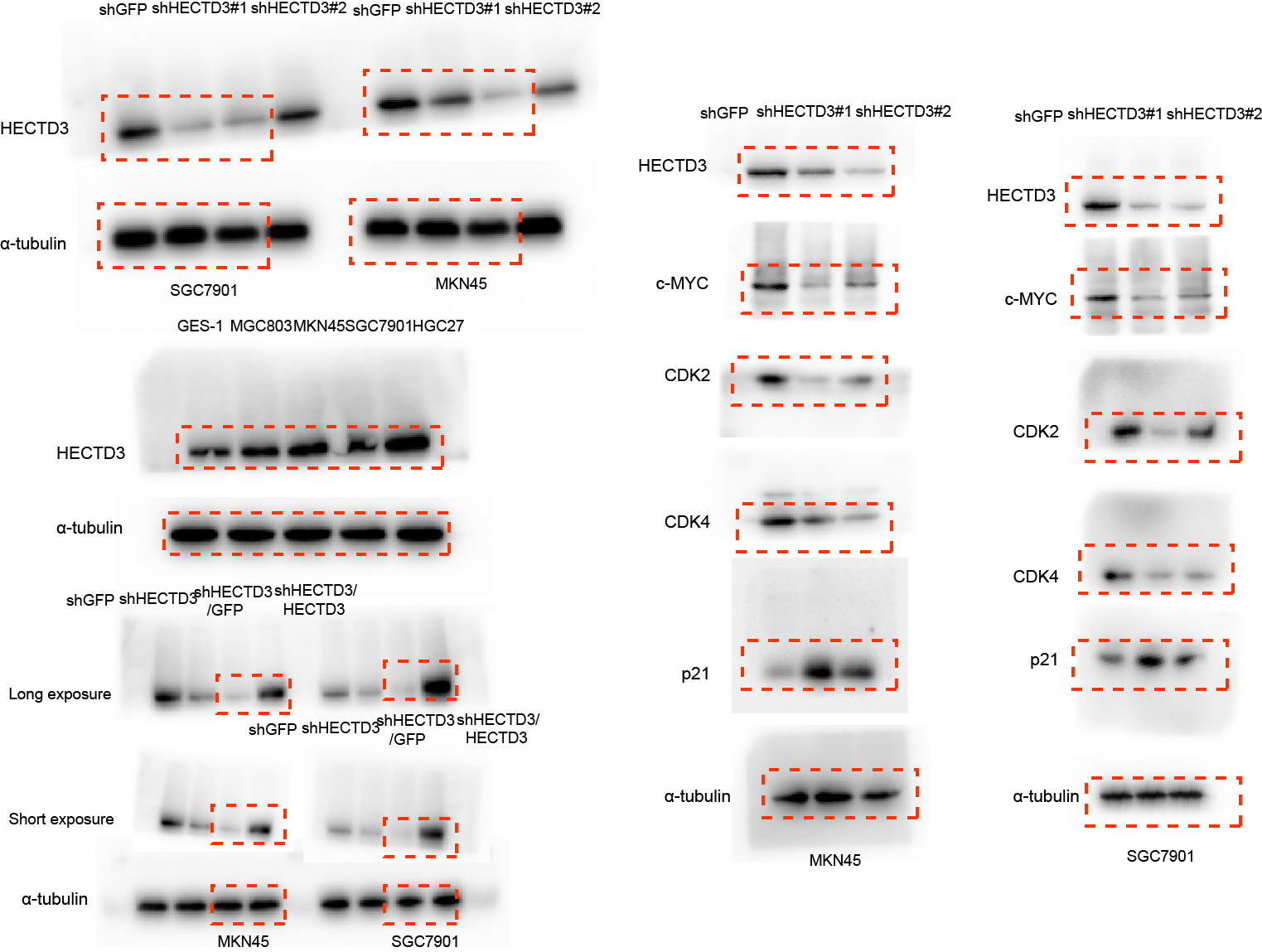


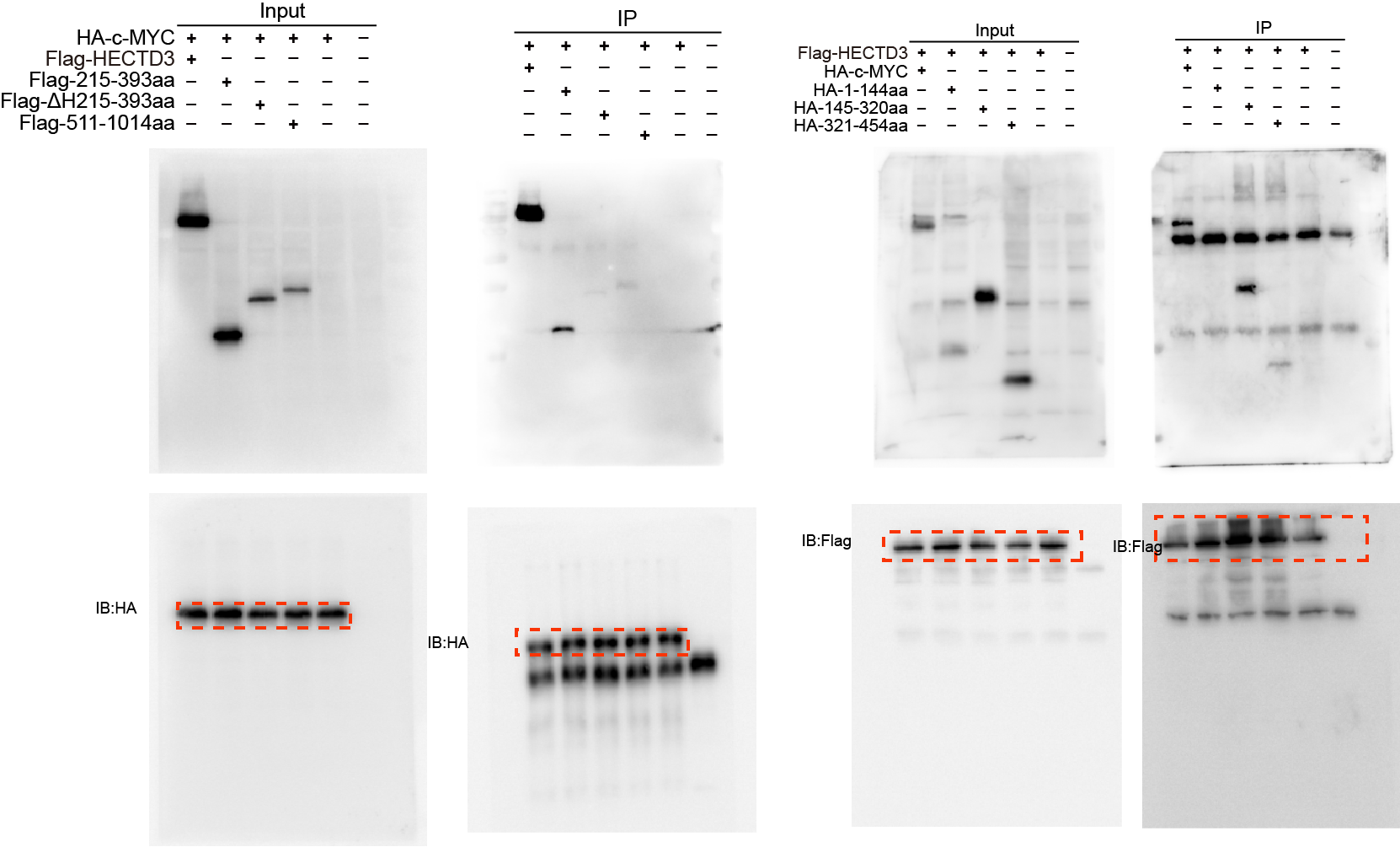


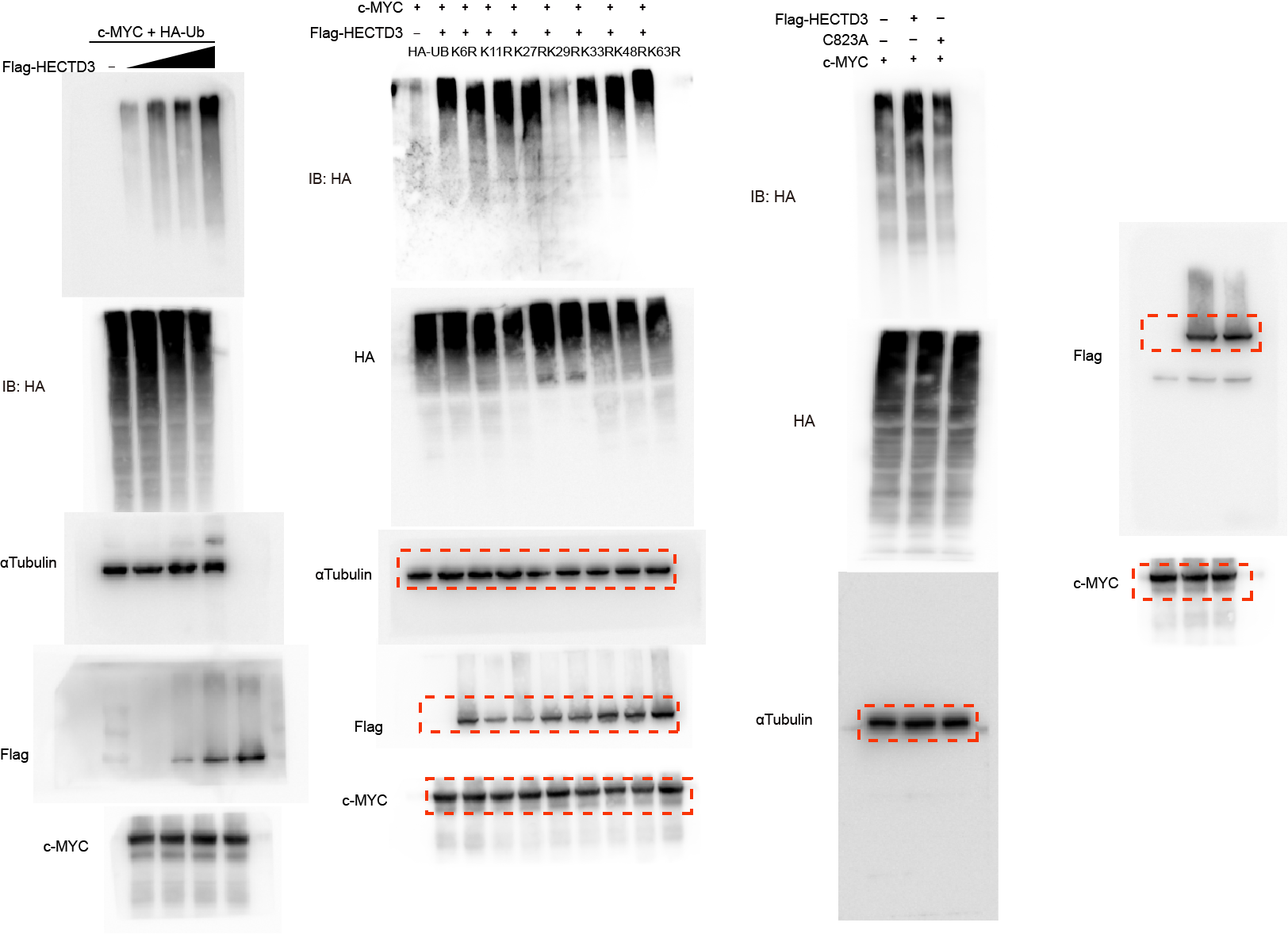


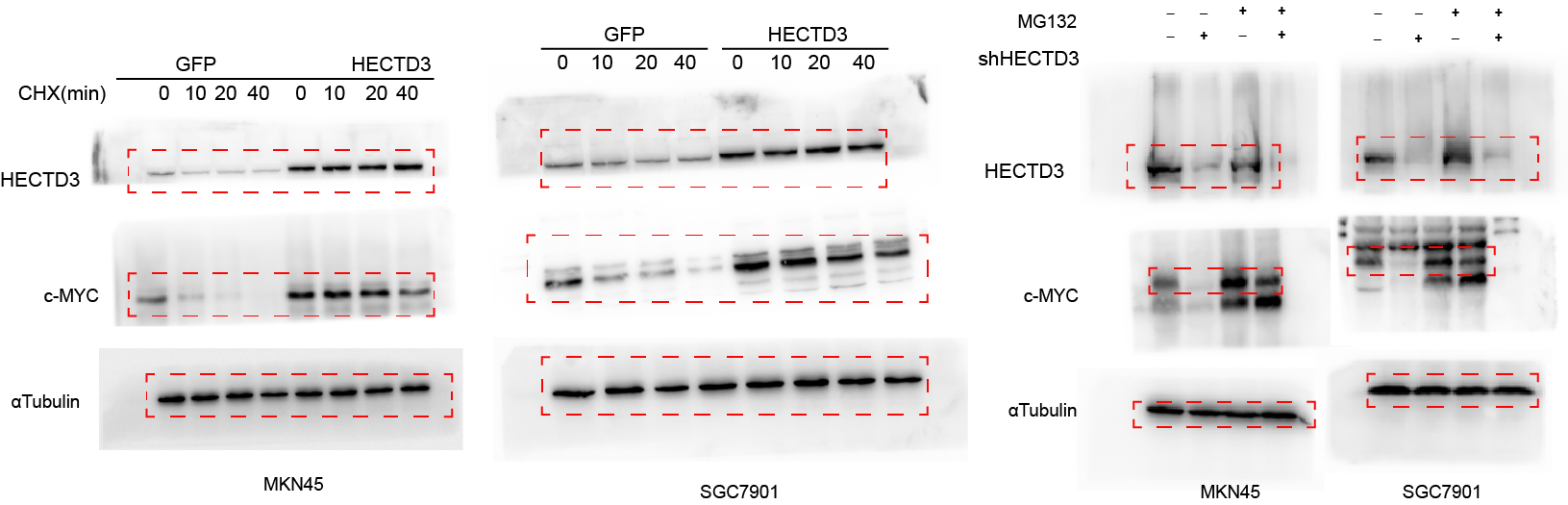

Supplement: Supplementary file 11 — Raw data-Western blot [file 41420_2022_1001_MOESM11_ESM.docx]

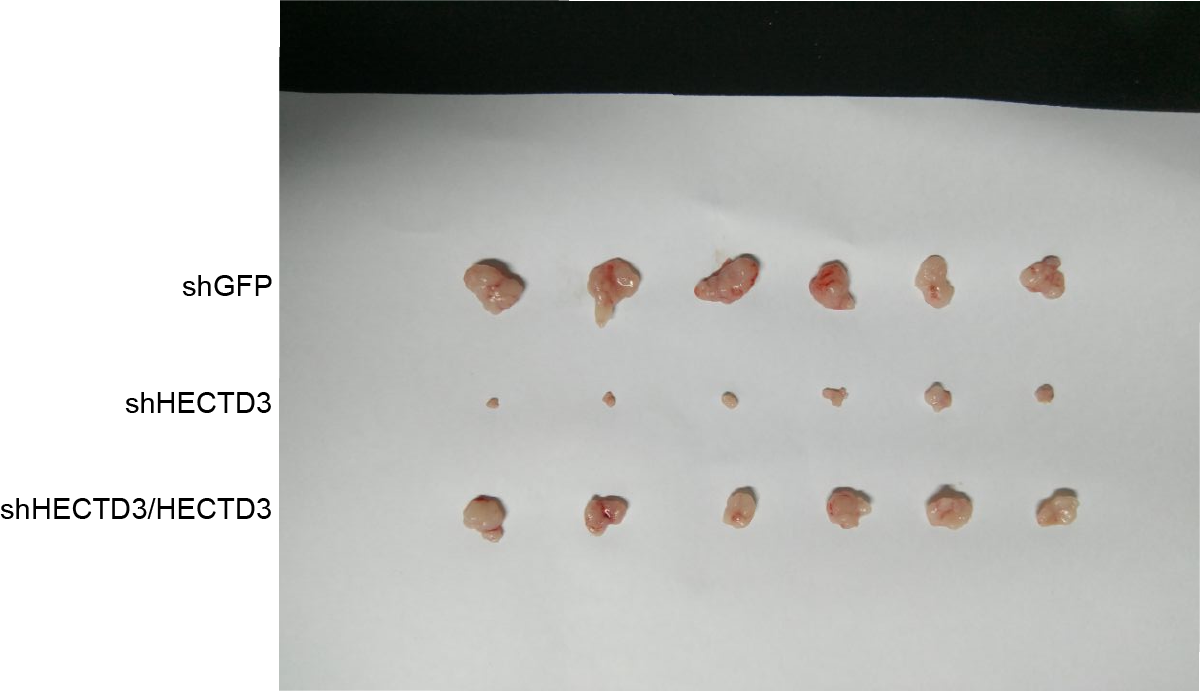


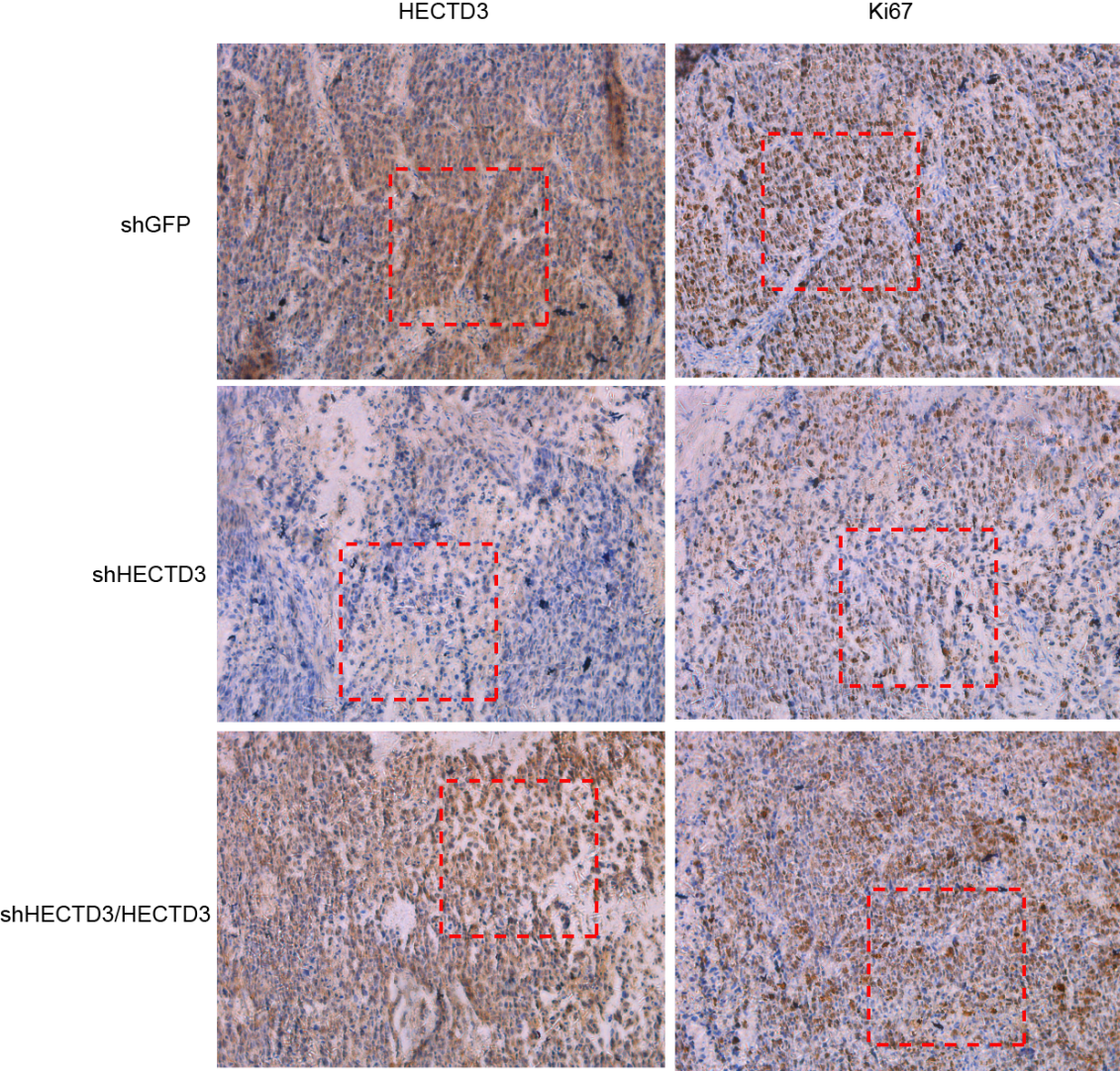

Supplement: Supplementary file 12 — Raw IHC and Xenograft experiment [file 41420_2022_1001_MOESM12_ESM.docx]
