# Supplementary material for: HECTD3 promotes gastric cancer progression by mediating the polyubiquitination of c-MYC
Source: Cell Death Discov. 2022 Apr 9;8:185. doi: 10.1038/s41420-022-01001-9 (PMC8994759; doi:10.1038/s41420-022-01001-9)
Supplement: Supplementary file 4 — Supplementary Figure notes [file 41420_2022_1001_MOESM4_ESM.docx]

**Supplementary Fig.1** (A) The expression of HECTD3 in gastric cancer cell lines and noncancerous gastric mucosa cells was detected through western blot assay.

**Supplementary Fig.2** (A) The proximity ligation (PLA) experiments were applied to assess the interaction of HECTD3 and c-MYC.

**Supplementary Fig.3** (A, B) Cycloheximide (CHX) was added to overexpressed HECTD3 cells to detect the degradation rate of c-MYC (C) The mRNA of c-MYC was detected through fluorescence quantitative PCR. (D) Proteasome inhibitor MG132 was added to gastric cancer cells in knockdown HECTD3 cells to detect the protein expression of c-MYC.
