# Supplementary material for: HECTD3 promotes gastric cancer progression by mediating the polyubiquitination of c-MYC
Source: Cell Death Discov. 2022 Apr 9;8:185. doi: 10.1038/s41420-022-01001-9 (PMC8994759; doi:10.1038/s41420-022-01001-9)
Supplement: Supplementary file 5 — Supplementary Table [file 41420_2022_1001_MOESM5_ESM.docx]

**shRNA and Fluorescence quantitative PCR primers Table**

| **ShHECTD3-Forward-1(5′−3′)** | **CCGGGCAGTCTTCACCCAGGTATATCTCGAGATATACCTGGGTGAAGACTGCTTTTTG** |
| --- | --- |
| **ShHECTD3-Reverse-1(5’-3’)** | **AATTCAAAAAGCAGTCTTCACCCAGGTATATCTCGAGATATACCTGGGTGAAGACTGC** |
| **ShHECTD3-Forward-2(5′−3′)** | **CCGGAGCTCTTTGGCGTGGATTATCCTCGAGGATAATCCACGCCAAAGAGCTTTTTTG** |
| **ShHECTD3-Reverse-2(5’-3’)** | **AATTCAAAAAAGCTCTTTGGCGTGGATTATCCTCGAGGATAATCCACGCCAAAGAGCT** |
| **RT-PCR-HECTD3-Forward** | **GGTGAAGCTGACAAAGGAGCA** |
| **RT-PCR-HECTD3-Reverse** | **TCCCAGGCGATGTGAGTATGT** |
| **RT-PCR-c-MYC-Forward** | **AATAGAGCTGCTTCGCCTAGA** |
| **RT-PCR-c-MYC-Reverse** | **GAGGTGGTTCATACTGAGCAAG** |
| **RT-PCR-GAPDH-Forward** | **GGAGCGAGATCCCTCCAAAAT** |
| **RT-PCR-GAPDH-Reverse** | **GGCTGTTGTCATACTTCTCATGG** |
