# Supplementary material for: HECTD3 promotes gastric cancer progression by mediating the polyubiquitination of c-MYC
Source: Cell Death Discov. 2022 Apr 9;8:185. doi: 10.1038/s41420-022-01001-9 (PMC8994759; doi:10.1038/s41420-022-01001-9)
Supplement: Supplementary file 6 — cddiscovery-author-contribution-form [file 41420_2022_1001_MOESM6_ESM.pdf]

DECLARATION OF CONTRIBUTIONS TO ARTICLE

ADMC

Manuscript Number:	Journal Name:	
CDDISCOVERY-21-2708R2	*Cell Death Discovery*	(the 'Journal')

Proposed Title of the Contribution:

HECTD3 promotes gastric cancer progression by mediating the polyubiquitination of c-MYC (the 'Contribution')

Author(s):

Guanghui Zhang, Qingzong Zhu, Xiaomin Yan, Mingxin Ci, Erhu Zhao, Jianbng Hou, Sicheng Wan, Muhan Lü*, Hongjuan Cui* (the 'Authors')

For all *CDDiscovery* articles, each person named as an author in the published version must be able to show he or she has contributed substantially to the article.

Authorship credit should be based on 1) substantial contributions to conception and design, acquisition of data, or analysis and interpretation of data; 2) drafting the article or revising it critically for important intellectual content; and 3) final approval of the version to be published. Authors should meet conditions 1, 2 and 3.

Any person who cannot be shown to have made a substantial contribution to the article cannot be listed as an author in the final version. The name of any person who is deemed to have made a minor contribution can, however, appear in the Acknowledgments section of the article.

Please complete the table below to indicate the contributions of all named authors to the manuscript.

Author Full Name:	Specification of Contribution to the Manuscript:
Guanghui Zhang	Guanghui Zhang designed experiments, obtained and analyzed data, and writed the manuscript.
Qingzong Zhu	Qingzong Zhu obtained and analyzed data, and writed the manuscript.
Xiaomin Yan	Xiaomin Yan obtained and analyzed data, and revised the manuscript.
Mingxin Ci	Mingxin Ci obtained and analyzed data, and revised the manuscript.
Erhu Zhao	Erhu Zhao obtained and analyzed data, and revised the manuscript.
Jianbng Hou	Jianbng Hou obtained and analyzed data, and revised the manuscript.
Sicheng Wan	Sicheng Wan obtained and analyzed data, and revised the manuscript.
Muhan Lü	Muhan Lüobtained and analyzed data, and revised the manuscript.
Hongjuan Cui	Hongjuan Cui designed experiments and revised the manuscript.

Please complete the table below to indicate the contributions of all named authors to the figures.

Figure 1:

In Figure 1, GZ and HC analyzed the data and prepared panel A-C; XY and MC analyzed the data and prepared panel D-E.

Figure 2:

In Figure 2, QZ and EZ generated the Western blot and PCR data and prepared panel A and B;. GZ, JH and SW generated MTT and BrdU data and prepared panel C and D.

Figure 3:

In Figure 3, HC, ML and QZ generated the soft agar and Flow cytometry data and prepared panel A-D;. GZ and XY generated Flow cytometry and Western blot data and prepared panel F and G.

Figure 4:

In Figure 4, GZ, JH and SW generated Western blot and MTT data and prepared panel A and B. HC, ML and QZ generated the BrdU, soft agar and Flow cytometry data and prepared panel C-E

Figure 5:

In Figure 5, GZ and HC generated and analyzed the data and prepared panel A-C. GZ, JH and SW generated Western blot data and prepared panel D and E. HC, ML and QZ generated the Western blot and Ubiquitination data and prepared panel F-H.

Figure 6:

In Figure 6, QZ and EZ gerated the xenograft assay and prepared the panel A-C. HC, GZ and QZ generated immunohistochemical experiment data and drawn the mechanism diagram.

Signed for and on behalf of the Author(s):	Print Name:	Date:

Signed for and on behalf of the authors:	Print Name:	Date:
Guanghui Zhang	Guanghui Zhang	2022.1.11
Qingzong Zhu	Qingzong Zhu	2022.1.11
Xiaomin Yan	Xiaomin Yan	2022.1.11
Mingxin Ci	Mingxin Ci	2022.1.11
[illegible]	Erhu Zhao	2022.1.11
Jianbing Hou	Jianbing Hou	2022.1.11
Sicheng Wan	Sicheng Wan	2022.1.11
Muhan Lü	Muhan Lü	2022.1.11
Hongjuan Cui	Hongjuan Cui	2022.1.11
